# Identification of osteoporosis genes using family studies

**DOI:** 10.3389/fendo.2024.1455689

**Published:** 2024-10-22

**Authors:** Marichela Schembri, Melissa M. Formosa

**Affiliations:** ^1^ Department of Applied Biomedical Science, Faculty of Health Sciences, University of Malta, Msida, Malta; ^2^ Centre for Molecular Medicine and Biobanking, University of Malta, Msida, Malta

**Keywords:** genetics, osteoporosis, family studies, fragility fractures, high-throughput sequencing

## Abstract

Osteoporosis is a multifactorial bone disease characterised by reduced bone mass and increased fracture risk. Family studies have made significant contribution in unravelling the genetics of osteoporosis. Yet, most of the underlying molecular and biological mechanisms remain unknown prompting the need for further studies. This review outlines the proper phenotyping and advanced genetic techniques in the form of high-throughput DNA sequencing used to identify genetic factors underlying monogenic osteoporosis in a family-based setting. The steps related to variant filtering prioritisation and curation are also described. From an evolutionary perspective, deleterious risk variants with higher penetrance tend to be rare as a result of negative selection. High-throughput sequencing (HTS) can identify rare variants with large effect sizes which are likely to be missed by candidate gene analysis or genome-wide association studies (GWAS) wherein common variants with small to moderate effect sizes are identified. We also describe the importance of replicating implicated genes, and possibly variants, identified following HTS to confirm their causality. Replication of the gene in other families, singletons or independent cohorts confirms that the shortlisted genes and/or variants are indeed causal. Furthermore, novel genes and/or variants implicated in monogenic osteoporosis require a thorough validation by means of *in vitro* and *in vivo* assessment. Therefore, analyses of families can continue to elucidate the genetic architecture of osteoporosis, paving the way for improved diagnostic and therapeutic strategies.

## 1 Introduction

Osteoporosis is a progressive skeletal disorder characterised by low bone mass and degradation of the microarchitecture of bone tissue that subsequently decrease bone strength and increase susceptibility to fragility fractures, such as those occurring from standing height or less (1, 2). Osteoporosis is considered a ‘silent disease’ until the first fracture occurs (3). The most sustained fractures are those of the hip, spine, wrist and humerus, resulting in increased morbidity, need for hospital care and institutionalisation. Moreover, spine and hip fractures can also result in death due to secondary complications (4). Consequently, osteoporosis is associated with a significant burden on the global economy and healthcare system with costs expected to increase due to a rise in the ageing population. Globally, osteoporosis is estimated to affect one in three women and one in five men over the age of 50 years (5). The prevalence of osteoporosis in the United States in the years 2017-2018 was approximately 12.6% among adults aged 50 years and over (6). In 2019, 32 million people in the EU27 + 2 (European Union countries, Switzerland, and the United Kingdom) were reported to have osteoporosis (7).

Osteoporosis may arise due to an imbalance in the bone remodelling equilibrium with a bone resorption rate that exceeds bone formation, failure to reach peak bone mass at the young adult stage, and/or due to physiological changes occurring in the body with advancing age such as oestrogen deficiency (6). All three scenarios are influenced by environmental (such as diet, smoking, alcohol intake and physical activity) and genetic factors (e.g., gender, low birth weight, early menopause, and low body mass index) (7). The risk exerted by these factors varies, with genetic factors imposing one of the strongest effects. Indeed, twin and family studies have shown that 50-85% of the changes in the bone mineral density (BMD) are genetically determined (8, 9). Genome-wide association studies (GWAS) have identified several gene variants as potential contributors of bone mass determination and fracture risk, each exerting modest effect sizes. In contrast, monogenic forms of osteoporosis are caused by a single gene variant that plays a significant role in skeletal development. Family studies harbouring multi-generation, affected relatives have aided in the identification of high-impact disease-causing genes and variants, some of which replicated at the population level. This brief review aims to provide an overview of the promising application of a family-based study approach to uncover the underlying genetic determinants of osteoporosis following high-throughput sequencing (HTS).

## 2 Family-based study design

### 2.1 Benefit of families in genetic study approaches

Family-based studies have successfully been used to identify genes underlying a variety of monogenic, highly penetrant disorders such as Duchenne muscular dystrophy (OMIM: 310200) (10, 11), Huntington disease (OMIM: 43100) (12), and cystic fibrosis (OMIM: 219700) (13). Generally, complex traits are differentiated from monogenic disorders in that: (i) they are more prevalent; (ii) do not demonstrate clean mendelian segregation patterns suggesting a polygenic cause whereby multiple gene variants (possibly involved in different signalling cascades) having different effect sizes collectively contribute to the phenotype; and (iii) the marginal effect of any single gene on a relevant clinical end point is likely to be small (14). The use of family studies having multiple affected members overcomes such issues. Identifying genes through family studies serves as a foundation in population-based research, addressing the various challenges inherent in analysing the genetic makeup of complex traits (15–18).

**Figure 1:**
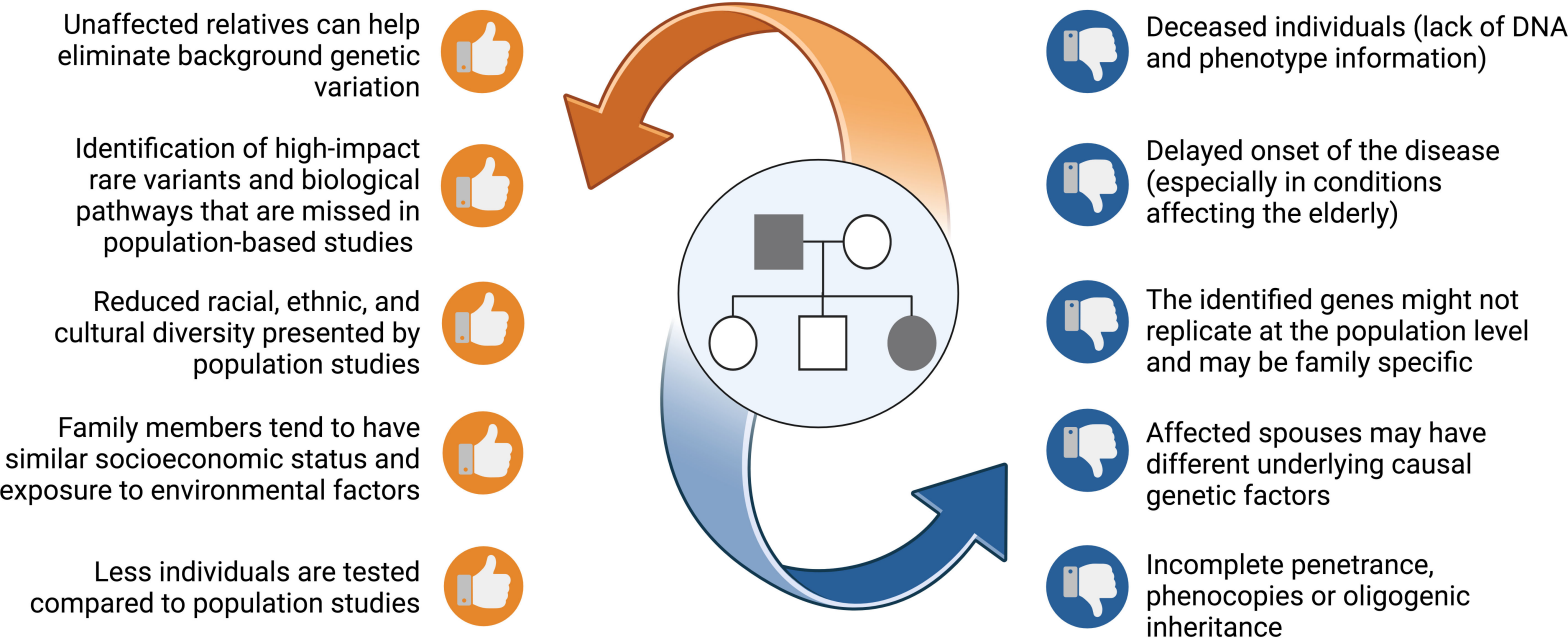
A list of benefits and risks that families offer when pursing genetic studies. Figure created using BioRender.

Family studies offer several benefits for gene discovery as opposed to studies of unrelated individuals (**Figure 1**). This rests on the fact that family members are more likely to possess a homogenous and limited set of causative genes. Therefore, the statistical power for gene discovery is enhanced and findings can be replicated across other affected families. Compared to population studies, unrelated or related but unaffected relatives serve as good controls, most noticeably since they share common environmental influences and have a similar socioeconomic status with affected relatives. In addition, unaffected relatives share a good proportion of their genes, that can be used to control background genetic variation shifting the focus to potential causal variants (15, 18). Despite being unbiased, this approach requires correct and extensive pedigree information, along with good phenotyping and a distant relative or control to help in variant filtering.

### 2.2 Successful use of family-based studies in bone diseases

Prior to the completion of the human genome project, highly penetrant variants with large effect sizes were identified using linkage analysis in families with monogenic bone disorders. This approach is based on the principle of identity-by-descent in combination with phenotypic information to identify the common gene loci shared amongst affected family members. The biological phenomenon of linkage analysis is that the closer the genetic marker is to the causative gene, the less likely it is to be separated by meiotic recombination between generations (19). Linkage analysis studies have identified a number of genes responsible for rare monogenic bone disorders including sclerosteosis (*SOST*; OMIM: 605740) (20), van Buchem disease (*SOST*; OMIM: 605740) (21), osteogenesis imperfecta (OI) including type I, II, III and IV (*COL1A1*; OMIM: 120150 and *COL1A2*; OMIM: 120160) (22, 23), osteoporosis-pseudoglioma syndrome (*LRP5*: OMIM: 603506) (24), and osteopetrosis (*CLCN7*: OMIM: 602727) (25). These genes have provided a better understanding of bone pathophysiology, some of which have paved the way for the development of targeted osteoanabolic therapy such as in the case of Romosozumab (26).

The cost effectiveness of HTS, coupled with its faster turnaround time, increased access, and wealth of data generated has led to a shift in the type of genetic testing undertaken, with omics technology favoured over classical linkage analysis and Sanger sequencing. HTS can be used to investigate genetic variation within exons (whole exome sequencing; WES), targeted genes (specific genes forming part of a panel that are known to contribute to disease) or the entire genome (whole genome sequencing; WGS). Short-read HTS has been instrumental in unravelling the genes underlying monogenic osteoporosis, reviewed in detail elsewhere (27–29). Independent family studies followed by WES have identified variants in the *WNT1* (OMIM: 164820) involved in early-onset osteoporosis (EOOP) and OI type III (30, 31), and *PLS3* (OMIM:30013) in four Dutch families with X-linked osteoporosis, with a specific *PLS3* variant successfully replicating at the population level (32). Several other causal *PLS3* mutations have since been reported in EOOP, including missense, nonsense and structural variants (SVs) (33–35). HTS has also identified two novel loss-of-function mutations in *LRP5* (OMIM: 603506) that provided further knowledge on the role of this gene in canonical WNT signalling and its effect in the development of juvenile-onset primary osteoporosis (OMIM: 619884) (36). Besides osteoporosis, *LRP5* variants have been implicated in high bone mass disorders and have also achieved genome-wide significance with hip and spine BMD (37–39). Other studies using a family-based approach combined with HTS have successfully identified genes with a clear-cut role in bone physiology including, *SGMS2* (OMIM: 611574) (40), *ARHGAP25* (OMIM: 610587) (41), *WNT11* (OMIM: 603699) (42), *RUNX1* (OMIM: 151385) (43), and *FGFR2* (OMIM: 176943) (44). Besides osteoporosis, this study design has also been used to uncover genetic determinants in other diseases such as Alzheimer’s disease (45), Parkinson’s disease (46), diabetes mellitus (47–49) and cardiovascular disease (50, 51).

## 3 Gene discovery using family studies

**Figure 2:**
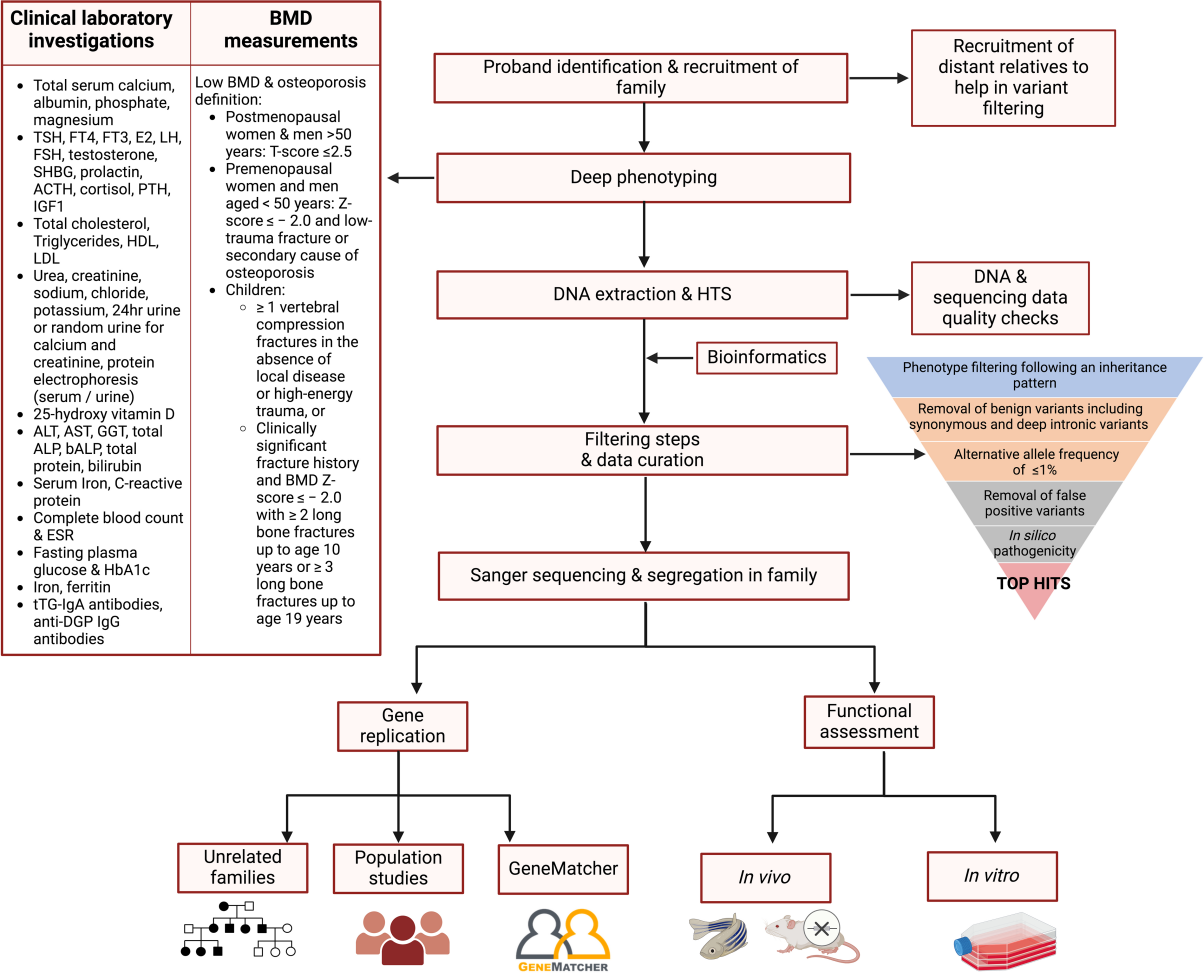
Proposed flowchart for identifying the genetic variants contributing to osteoporosis in a family study. Affected proband and relatives are recruited and subjected to deep phenotyping followed by genetic evaluation using HTS. A stepwise filtering scheme is applied with the aim of filtering out common, benign, and low-penetrance variants that are unlikely to be causal, retaining high-impact variants possibly residing in genes having a role in bone biology. Segregation of the variants in the entire pedigree is confirmed by Sanger sequencing. The shortlisted variants are tested in other independent families and/or population studies, and functionally validated using cells or animal models to confirm their role in bone metabolism and osteoporosis pathogenesis. ACTH, adrenocorticotropic hormone; ALP, alkaline phosphatase; ALT, alanine transaminase; AST, aspartate transaminase; DGP, deamidated gliadin peptide; E2, oestradiol; ESR, erythrocyte sedimentation rate; FSH, follicle-stimulating hormone; FT3, free T3; FT4, free T4; GGT, gamma-glutamyltransferase; HbA1c, glycosylated haemoglobin; HDL, high-density lipoprotein; IGF1, insulin-like growth factor 1; LH, luteinising hormone; LDL, low-density lipoprotein; PTH, parathyroid hormone; SHBG, sex hormone-binding globulin; TSH, thyroid-stimulating hormone; tTG, tissue transglutaminase; 25-OH Vitamin D, 25-hydroxy vitamin D. Figure created using BioRender.

The following steps are recommended to identify rare, high-risk DNA variants in individuals with suspected monogenic osteoporosis (**Figure 2**): recruitment of family members, rigorous deep phenotyping, genetic evaluation of HTS data including variant filtering and prioritisation, co-segregation of variants in the entire pedigree, and if possible, detection of the deleterious gene and/or variant in other families and/or population studies.

### 3.1 Recruitment of family members

Maximising the inclusion of both affected and unaffected individuals in multigenerational pedigrees enhances the likelihood of identifying causal variants. Affected family members should be directly related by blood (at least one affected relative in each generation), with a minimal number of affected spouses. A thorough clinical history for all recruited individuals is crucial and should involve data concerning demographic and lifestyle factors such as physical activity, calcium and Vitamin D intake, alcohol consumption, smoking habits, age at menopause and medication use. A comprehensive fracture history should comprise of all fractures sustained from childhood and specify the age at which each fracture occurred, the site of fracture and the mechanism of fracture (low, moderate, or high-impact trauma). Family history of bone disease, osteoporosis and low-trauma fractures is also essential, irrespective of age.

### 3.2 Phenotyping of family members

Precise differentiation between affected and unaffected relatives is crucial, as incorrect phenotyping can hinder the identification of causal gene variants. Laboratory analyses should include relevant biochemical parameters of coexisting diseases that may affect bone homeostasis (52–54). Furthermore, anthropometric measurements should also be recorded using validated equipment which is calibrated according to WHO guidelines (55). Dual-energy X-ray absorptiometry (DXA) remains the gold standard for the non-invasive measurement of BMD in all age groups, including children and young adults thanks to its worldwide availability, precision, reproducibility, and availability of normative data (54). BMD measurements are expressed in T-scores and Z-scores based on the WHO classification and the International Society for Clinical Densitometry (ISCD) for ease of interpretation (56). An affected status may be defined by a T-score of ≤-2.5 in the case of postmenopausal women and men over the age of 50; and a Z-score below -2.0 accompanied by a low-trauma fracture history in the case of premenopausal women and men younger than 50 years (55). In the case of EOOP, an affected status in children and young adults is further defined in (29).

### 3.3 Genetic analysis and bioinformatics

Genomic DNA is typically isolated from peripheral blood leukocytes using ethylenediaminetetraacetic acid (EDTA) or citrated blood tubes or harvested buffy coat layers depending on blood volumes available. Where possible, aliquots of whole blood (with or without stabilisers), serum and plasma samples should be banked (at -80°C) for potential future omics studies (i.e., transcriptomics, metabolomics, proteomics). If phlebotomy is not possible, genomic DNA can be obtained from saliva or buccal swabs. High-quality genomic DNA is essential for accurate and comprehensive short-read sequencing, whereas high-molecular weight DNA is required for long-read sequencing (57). HTS should be carried out on the most genetically informative relatives based on the structure of the pedigree. WGS is becoming an attractive alternative to WES due to its broader coverage and decreasing costs. Unlike WES, WGS does not require enrichment and capture steps, leading to a more uniform coverage and improved detection of variants (58). If no significant variants are identified in the extended coding regions, non-coding variants can be further scrutinised. Before analysing variants, sequencing data must undergo quality control to avoid losing statistical power, minimising false positives and negative results (59). FastQC can be utilised to perform quality checks on raw HTS data providing modular analyses including pre-base analysis of sequencing reads with the aim of pinpointing sequencing artefacts that may affect downstream analyses (60). Subsequently, clean raw reads are aligned to the latest version of the human reference genome, such as UCSC Genome (61) or NCBI RefSeq (62). The most commonly used aligners for short read sequencing data are Burrows-Wheeler Aligner (63), MOSAIK (64), and Bowtie (65). The aligned reads are run through Picard tools to flag any duplicate reads arising from enrichment bias during sequencing. Tools such as FastQC (60) and fastp (66) can be used for duplicate removal. The following step is variant calling whereby aligned reads are compared to the reference genome and any nucleotide variations are identified. SNV (single nucleotide variants) and InDel (Insertions or Deletions) calling can be performed using specific tools such as Genome Analysis Tool Kit HaplotypeCaller (GATK-HC) (67), Samtools mpileup (68), DeepVariant (69), and varScan (70). Calling of SVs can be done by running the BAM files in either LUMPY (71) or Manta (72), and in the case of copy number variations also using CNVnator (73) or CNVkit (74). VEP (Variant Effect Predictor) (75) and SnpEff (76) are commonly employed to annotate variants based on the genomic location, and to predict the functional effect on a gene. Additionally, variant calling tools incorporate information related to: (i) alternative allele frequency from public databases such as the 1000 Genomes Project (77), the Single Nucleotide Polymorphism Database (dbSNP) (78), and Genome Aggregation Database (gnomAD) (79); (ii) conservation scores according to PhyloP (80) and the Genomic Evolutionary Rate Profiling (GERP) score (81); and (iii) *in silico* predictions of variant pathogenicity according to Polymorphism Phenotyping (PolyPhen-2) (82), Sorting Intolerant From Tolerant (SIFT) (83), The Likelihood Ratio Test (LRT) (84), Variant Effect Scoring tool (VEST3) (85), MutationTaster (86), MutationAssessor (87), MetaLR (88), Functional Analysis through Hidden Markov Models (FATHMM) (89), and Meta-analytic support vector machine (MetaSVM) (90), amongst others.

### 3.4 Variant filtering, prioritisation and curation

Based on a thorough literature review, the following filtering pipeline is recommended to narrow down the extensive list of gene variants, leaving a shortlist of potentially causal variants as described below.

- Phenotype filtering following an inheritance pattern as observed for the studied family can help remove the shared benign genetic variants shifting the focus on causal gene variants.
- Removal of low impact variants including synonymous, as well as deep intronic and intergenic variants, in favour of nonsense (stopgain) and stoploss variants, missense, frameshift (including those in splicing regions or resulting in start/stop loss), and variants affecting splice sites (within 20 nucleotides upstream or downstream of exon-intron boundaries).
- Retaining of variants with an observed alternative allele frequency (AAF) of ≤1% in all population-based allele frequency databases, particularly gnomAD for SNVs, InDels and more recently also SVs, assuming the presence of rare and penetrant variants.
- Removal of recurrent false positive variants (also known as ‘frequent hitters’) resulting from assembly misalignment, variants falling in highly polymorphic areas and mislabelled variants due to misleading reference genome data (91, 92).
- Prioritisation of variants based on *in silico* pathogenicity, Combined Annotation–Dependent Depletion (CADD) score (93), conservation scores, and classification by the American College of Medical Genetics and Genomics/Association for Molecular Pathology (ACMG/AMP) guidelines (94). Various *in silico* tools, particularly those for coding variants, exhibit different thresholds and cut-offs for variant scoring and have variable concordance with each other. Consequently, it is advised to utilise multiple *in silico* tools concurrently for variant interpretation, and a scoring criterion to ensure consistency. Variants predicted to be ‘deleterious’/’pathogenic’/’damaging’ by most of the tools will be given priority over variants predicted to be ‘benign’/’neutral’/’tolerated’ (95). VarSome (96) should also be used to further assess the impact of variants on protein structure and function. This tool is based on an accurate analysis of HTS data from numerous databases such as MetaRNN (97), DANN SNVs (98), UniProt (99), dbscSNV (100), gnomAD and ClinVar (101). Moreover, VarSome automatically performs the classification of genetic variants according to the ACMG/AMP guidelines.
- To further curate the variant list, those residing in genes involved directly or indirectly in bone physiology or expressed in bone tissue can be prioritised. Some examples of resources and online databases which can be utilised include: Mouse Genome Informatics (http://www.informatics.jax.org) (102), the Musculoskeletal Knowledge Portal (http://mskkp.org/) (103), International Mouse Phenotyping Consortium (https://www.mousephenotype.org/) (104), Gene Ontology (http://www.geneontology.org) (105), Online Mendelian Inheritance in Man (http://omim.org) (106), HumanBase network (https://hb.flatironinstitute.org/) (107), Kyoto Encyclopaedia of Genes and Genomes (KEGG) Pathway (available at http://www.genome.jp/kegg/) (108), and QIAGEN’s Ingenuity Pathway Analysis^®^ (109).

### 3.5 Confirmation of shortlisted variants and co-segregation studies

All remaining shortlisted variants should be analysed in the IGV software (Integrative Genomics Viewer, Broad Institute and the Regents of the University of California, USA) (110) for the distinction between true variants and false-positive hits caused by misalignments or inaccurate bioinformatics processing results. Moreover, IGV is used to compute the read coverage in the viewed region and allele ratios for the observed genotypes. Sanger sequencing can be used as a secondary approach to further verify the shortlisted variants. Furthermore, this method should also be used to determine the variants’ segregation across the non-sequenced relatives in the pedigree. Ideally, the shortlisted causal variant should only be present in the affected relatives. However, the consideration of unaffected carriers should not be excluded due to factors such as incomplete penetrance or the late onset of the disease (**Figure 1**). Oligogenic inheritance, characterised by the segregation of multiple variants within a pedigree, is also a plausible scenario.

### 3.6 Replication and validation of the shortlisted variants

The shortlisted variants can be tested in other affected families to provide further proof of disease gene causation. A genetic variant within the same potentially causal gene should be identified in at least two independent, unrelated probands or families having the same disease (or phenotype). This criterion helps establish a more robust association between the gene and the phenotype, reducing the likelihood that the association is coincidental. GeneMatcher (111) was specifically set up to enable clinicians and researchers to make such connections. Additionally, the shortlisted variant(s) or others residing within the identified deleterious gene should be sought in large collections of unrelated individuals to determine whether they associate with BMD and other musculoskeletal phenotypes at the population level. Finally, functional assessment using *in vitro* cells and *in vivo* (e.g., mice or zebrafish) models (**Figure 2**), as well as expression studies, are warranted to understand the gene and/or variant’s biological role in bone and osteoporosis pathogenesis. The identification of a high impact, loss-of-function variant can be considered analogous to a “human knockout” for that gene. The shortlisted variant, the mode of inheritance of the disease, gene expression levels, and the functional characteristics of the associated protein should all be taken into consideration when designing a functional study, described further elsewhere (28, 112).

## 4 Discussion: limitations and future work

While family-based studies are a powerful tool in genetic research, there are several pitfalls that one must consider, including the presence of phenocopies and incomplete penetrance (**Figure 1**). Integrating additional methodologies, such as long-read sequencing (that is better suited to capture large SVs), multi-omics studies, and advanced computational models (e.g., machine-learning tools) (113), can help overcome some challenges leading to a more comprehensive understanding of the genetic and phenotypic relationships. In fact, the integration of genomic and metabolomic data provided further proof of the role of *SGMS2* in osteoporosis and skeletal dysplasia (40). A multi-omics approach can provide a holistic integrated view from a system biology perspective, capturing the complexity of the underlying pathological mechanisms, and presenting opportunities for biomarker discovery. Furthermore, the importance of a multidisciplinary team involving clinical, basic and translational researchers, and bioinformaticians is becoming more evident for improved patient care with timely diagnosis and optimal treatment options. Active collaboration in international scientific consortia (e.g., GEFOS and GENOMOS), European Reference Networks (e.g., European Network for Rare Bone Conditions, ERN BOND), disease registries and patient organisations is the way forward.

## 5 Conclusion

As highlighted in this review, family-based studies have been instrumental in identifying genetic determinants governing bone metabolism and disease processes giving rise to osteoporosis and other bone mass disorders. To date, around 20% of the underlying genetic factors are known, emphasising the need for further research efforts in the field (114). Genes and variants uncovered in family studies may lead to the development of diagnostic biomarkers and drug targets based on an individual’s genetic make-up. This makes personalised medicine more of a reality, which is the ultimate goal of genomic studies.
